# In the moral eye of the beholder: the interactive effects of leader and follower moral identity on perceptions of ethical leadership and LMX quality

**DOI:** 10.3389/fpsyg.2015.01126

**Published:** 2015-08-04

**Authors:** Steffen R. Giessner, Niels Van Quaquebeke, Suzanne van Gils, Daan van Knippenberg, Janine A. J. M. Kollée

**Affiliations:** ^1^Rotterdam School of Management, Erasmus UniversityRotterdam, Netherlands; ^2^Kuehne Logistics UniversityHamburg, Germany; ^3^Faculty of Psychology and Neuroscience, Maastricht UniversityMaastricht, Netherlands

**Keywords:** moral identity, ethical leadership, LMX, follower perspective, leader perspective

## Abstract

Previous research indicated that leader moral identity (MI; i.e., leaders’ self-definition in terms of moral attributes) predicts to what extent followers perceive their leader as ethical (i.e., demonstrating and promoting ethical conduct in the organization). Leadership, however, is a relational process that involves leaders and followers. Building on this understanding, we hypothesized that follower and leader MI (a) interact in predicting whether followers will perceive their leaders as ethical and, as a result, (b) influence followers’ perceptions of leader–follower relationship quality. A dyadic field study (*N* = 101) shows that leader MI is a stronger predictor of followers’ perceptions of ethical leadership for followers who are high (vs. low) in MI. Perceptions of ethical leadership in turn predict how the quality of the relationship will be perceived. Hence, whether leader MI translates to perceptions of ethical leadership and of better relationship quality depends on the MI of followers.

## Introduction

Recent business scandals have drawn public and academic attention to the question of how moral our business leaders actually are. Aquino and Reed (2002) proposed that moral behaviors are at least partly predicted by individual self-definition in terms of moral attributes which they call *moral identity* (MI). Individuals with a stronger MI are assumed to be more inclined to regulate their own behavior in terms of its morality (Aquino et al., 2009). As a result, leaders with a higher MI are perceived as more ethical by their followers (Mayer et al., 2012a) – a perception that ultimately also results in better quality leader–follower exchange (LMX) relationships (Mahsud et al., 2010; Tumasjan et al., 2011).

Leadership is a process of social influence between leaders and followers (Hollander, 1964; Bass, 1985; Graen and Scandura, 1987), and ethical leadership is no exception (Brown and Treviño, 2006). However, previous research has not considered followers’ own moral self-concept, i.e., their MI, as a boundary condition. More precisely, in order to understand what guides follower perceptions of ethical leadership and, consequently, the quality of LMX, we propose that one has to consider the interplay between leaders and followers (van Gils et al., 2010; Hernandez and Sitkin, 2011). In this study, we argue that perceptions of ethical leadership lie to a considerable extent in the eye of the beholder and, thus, also depend on the MI of followers. In other words, we suggest that follower MI plays a crucial moderating role in the relationship between the MI of the leader and perceptions of ethical leadership.

Our research extends previous research in two important ways. First, we nuance the somewhat simplistic view that leaders who have a strong moral conviction (i.e., MI; and hence show ethical behavior) will also be automatically perceived as ethical leaders by their followers. Specifically, our central extension to previous theorizing is the argument that ethical behavior first has to be recognized as such by followers before the attribution of ethical leadership can follow. Whether followers can perceive respective leader behavior and hence label the leadership as ethical, so we argue, depends on the degree to which followers’ own MI is developed. Secondly, we reason that working with a leader who one perceives to be ethical is conducive to a better relationship with that leader. This perspective not only enriches theorizing within the domain of ethical leadership by outlining that the relationship quality between leaders and followers may be another effect of ethical leadership, but it also informs the wider LMX literature by suggesting that perceived morality may be an understudied component that feeds into the leader follower relationship quality.

### Leader Moral Identity and Ethical Leadership Perceptions

Moral identity is conceptualized as “a self-conception organized around a set of moral traits” (Aquino and Reed, 2002, p. 1424) such as being honest, caring, and compassionate. This definition is based on social cognitive theories of the self (cf. Kihlstrom and Klein, 1994). It is argued that individuals differ in the degree to which MI is central to their self-definition (Lapsley and Lasky, 2001; Aquino and Reed, 2002). This implies that the moral self-schema varies in its chronic accessibility (Lapsley and Lasky, 2001), and, as a result, this moral sense of self is more likely to be salient (i.e., cognitively activated) for persons with stronger chronic accessibility. As such, MI is one of many social identities to make up an individual’s self-definition (Markus, 1977; Tajfel and Turner, 1986; Aquino and Reed, 2002). Importantly, however, because MI consists of moral traits, it is conceptualized as an individual difference variable (i.e., in contrast to identities rooted in more transient group memberships) and represents a moral character perspective (Narvaez and Lapsley, 2009). More precisely, this perspective prescribes that specific moral traits are part of an identity schema that, in turn, prescribes one’s own behavior and serves as a guideline for judgments of others’ behavioral patterns.

Research on MI has distinguished two dimensions of MI. MI internalization refers directly to how important moral characteristics are to the self, while MI symbolization relates to the moral self as a social object which individuals can use to convey to others that they have these characteristics (Aquino and Reed, 2002). Together, these two dimensions reflect a person’s MI (Mayer et al., 2012a). However, as moral acts are generally thought to originate and be self-regulated from within (Rest, 1986; Bandura, 1999; Reynolds and Ceranic, 2007), the internalization dimension might reflect the core definition of MI more directly (Reynolds and Ceranic, 2007). Indeed, previous empirical research has consistently shown that the internalization dimension is a stronger predictor of moral behavior (Aquino and Reed, 2002; Reed and Aquino, 2003; Aquino et al., 2007; Reynolds and Ceranic, 2007). Further, Reed and Aquino (2003) argue that the symbolization dimension may mainly concern public behavior (i.e., behavior in the presence of a larger audience). Given that powerful actors such as leaders seem to be more immune to situational pressure and act more upon their internalized value orientation (Galinsky et al., 2008), we assume that the actions of leaders in dyadic relationships with their followers are driven more by the internalization dimension. Therefore, when we refer to MI below, we refer mainly to effects that are driven by the internalization dimension of MI.

Moral identity exerts its influence on individual behavior via the need for self-consistency (Blasi, 1983) and the related tendency of individuals to act consistently with their self-schemas. Thus, MI will act as a self-regulatory mechanism to the extent that it is central to an individual’s self-definition (Aquino and Reed, 2002). This social cognitive conception of MI has proven to be a central predictor of moral affect, moral cognition, and moral behaviors (see Shao et al., 2008, for a review). For instance, MI predicts pro-social behaviors such as making donations (Aquino and Reed, 2002; Reynolds and Ceranic, 2007) and showing moral regard to outgroup members (Reed and Aquino, 2003). Further, it relates negatively to unethical behaviors such as lying (Aquino et al., 2009) and cheating (Reynolds and Ceranic, 2007). Whereas most of this research was conducted in the field of psychology, the concept is attracting increasing attention in the management field (Detert et al., 2007; Reynolds and Ceranic, 2007).

Moral identity should also be an important variable to consider in leadership research – especially when studying the ethical behavior of leaders. Only relatively recently have researchers begun to understand the potential effects ethical leadership might have on the workforce (Treviño et al., 2003; Brown and Treviño, 2006; Detert et al., 2007; De Hoogh and Den Hartog, 2008; Neubert et al., 2009; Piccolo et al., 2010; Kalshoven et al., 2011). Our conceptualization of ethical leadership follows that of Brown et al. (2005, p. 120), who define it as “the demonstration of normatively appropriate conduct through personal actions and interpersonal relationships, and the promotion of such conduct to followers through two-way communication, reinforcement, and decision-making.” As MI is assumed to self-regulate human behavior through the need for self-consistency (Aquino and Reed, 2002), leaders with a stronger sense of MI should also display actions that reflect their morality. As a result, they should be perceived by their followers as displaying more ethical leadership and should motivate those followers to perform better (Mayer et al., 2012a). Based on our discussion above, we assume that the internalization dimension of MI should be the key driver of this relationship, because leaders should (a) be more immune to situational demands that influence more symbolic actions, (b) show actions that are driven primarily by an internalized value system (Galinsky et al., 2008), and also because (c) previous research has indicated that internalization (vs. symbolization) is overall a more reliable predictor of actual leader behavior (Reed and Aquino, 2003; Reynolds and Ceranic, 2007; Mayer et al., 2012a).

### Leader Moral Identity, Ethical Leadership Perceptions, and LMX Quality

Despite the fact that other researchers have shown that perceptions of ethical leadership improve LMX (Mahsud et al., 2010; Tumasjan et al., 2011), the full mediating relationship from leader MI via ethical leadership perceptions on LMX has remained uninvestigated. LMX theory has been the dominant theory, and focuses on how relationships between leader and follower develop and on the consequences of the quality of these relationships (Dansereau et al., 1975; Graen and Scandura, 1987; Graen and Uhl-Bien, 1995; Gerstner and Day, 1997; Dulebohn et al., 2012). LMX relationships develop through processes of tangible and intangible exchanges between leader and follower. For instance, a leader might provide information and support to the follower who, in return, performs tasks well and shows loyalty to the leader (Martin et al., 2010). These LMX relationships are assumed to be on a continuum from low-quality (i.e., relationships that are defined only by the employment contract) to high-quality (i.e., relationships that go beyond the formal job contract). These high-quality LMX relationships are characterized by mutual respect, trust and mutual obligations (Graen and Uhl-Bien, 1995; Martin et al., 2010). LMX theory suggests that the more leaders and followers develop relationships of this kind, the more effective leadership will be (Graen and Uhl-Bien, 1995; Anand et al., 2011). Further, it also proposes that each leader–follower dyad within a team is unique and can vary in quality, and that such LMX relationships must therefore be studied at the dyadic level. High-quality LMX was originally argued to be influenced primarily by the quality of the social exchange between leader and followers, but a range of empirical findings indicate that various antecedents beyond social exchanges predict the relationship quality (Martin et al., 2010; Dulebohn et al., 2012).

Follower perceptions of ethical leadership predict their perceptions of LMX quality for two reasons (Mahsud et al., 2010; Tumasjan et al., 2011). First, if leaders are perceived as ethical because of their fair procedures and their moral behavior in relation to followers, followers can be expected to reciprocate by showing commitment to their leaders, causing higher-quality LMX to emerge (Brown et al., 2005; Erdogan et al., 2006). Second, ethical leadership implies the establishment of trusting relationships that beyond economic exchanges which should additional increase perceptions of high-quality LMX. Thus, perceptions of ethical leadership initiate a morality-based process of social exchange that goes beyond classical dyadic exchange behavior (Brown and Treviño, 2006; Tumasjan et al., 2011). Integrating these insights, we base our model on the idea that leader MI can improve the quality of the LMX relationship through the mediating effect of perceptions of ethical leadership.

### The Moderating Effect of Follower Moral Identity

Most leadership researchers tend to focus on the traits or behaviors of the leader in order to understand effective leadership (Ilies et al., 2007). A MI perspective on leadership has a similar point of view, because it is a character based conception of morality (Narvaez and Lapsley, 2009). This implies that leaders will have the same impact via their behavior on all followers. However, research on LMX has shown that leadership can be understood as a process involving specific leader–follower dyads; leadership outcomes can differ from follower to follower (Dansereau et al., 1975; Graen and Uhl-Bien, 1995; Gerstner and Day, 1997). Leadership research has consistently shown that how followers perceive leader behavior is colored by their own cognitive frame of reference (Lord and Maher, 1991; Epitropaki and Martin, 2005; Van Quaquebeke et al., 2011). This follower-centric approach to leadership argues that leadership is partly constructed in the minds of followers (Meindl et al., 1985). Previous research shows convincingly that the self-concept of followers plays a crucial role in determining their perceptions – and implicit theories – of leadership (Lord and Brown, 2004; Dinh et al., 2013).

This moderating process of follower perceptions should arguably hold also for leader MI as a predictor of ethical leadership perceptions (cf. van Gils et al., 2010; Jordan et al., 2013). There are two main arguments why follower MI should influence the relationship between leader MI (and the resulting behavioral patterns) and followers’ perceptions of ethical leadership. First, ethical leadership is defined as an interpersonal relationship between leaders and followers. Thus, the follower is an essential element to understand and describe ethical leadership. Second, ethical leadership is not defined in terms of character-based perspective, but rather as a descriptive concept. In other words, ethical leadership is a (perceived) normatively appropriate behavior of the leader and the reinforcement of such behavior (Brown et al., 2005). Therefore, leader behavior stemming from the MI schema such as being honest, caring, and compassionate should only lead to ethical leadership if it is deemed to be normative appropriate by the respective follower. Consequently, we argue that follower might vary in the degree to which they would perceive leader behavior based on the leader’s MI is normatively appropriate and, thus, ethical. We predict that individual differences in follower MI play a central moderating role in the relationship between leader MI and follower perceptions of ethical leadership.

Moral identity is understood as influencing not only moral actions but also moral cognitions. Because the concept of MI is rooted within the social identity perspective (Markus, 1977; Tajfel and Turner, 1986), it is defined as a social self-schema which directs attention to self-relevant information. While many social identities become more or less salient in different situations, MI is believed to be more chronically accessible as it is “deeply linked to a person’s self-conception” (Aquino and Reed, 2002, p. 1425). If MI is central to individual’s sense of self (i.e., high MI), it implies that this part of the self-schema will direct that individual to focus attention on relevant moral information. In other words, such people would generally “show greater concern for the welfare of others, being more socially responsible, [….] and having a greater social conscience” (Reed et al., 2007; p. 183). As a result, followers whose MI has high (vs. low) centrality are more likely to perceiver leader behaviors that reflect their own morality as normatively appropriate and, thus, as ethical leader behaviors. Put differently, a leader’s moral behaviors should (a) receive greater attention from and (b) provide more normative appropriateness in the ethical leadership evaluation process from followers with high MI (Reynolds, 2008; van Gils et al., 2014). Given that the internalization dimension of MI reflects how far moral traits are core to one’s self-concept (Reed and Aquino, 2003), we expect that this dimension will primarily influence followers’ perceptions of their leader’s behavior. Consequently, leader MI will have a stronger impact on followers’ perceptions of ethical leadership where those followers themselves are high in MI (internalization). In contrast, followers who are low in MI (internalization) have a self-concept that is less deeply linked to moral traits such as being caring, considerate, and friendly (Aquino and Reed, 2002). As a result, these followers are likely to pay less attention to leader behaviors that derive from the leader’s MI (internalization), and their ethical leadership perceptions will be less influenced by the specific leader behaviors resulting from the centrality of a leader’s MI.

H1: Follower MI (internalization) moderates the relationship between leader MI (internalization) and follower perceptions of ethical leadership; the stronger the MI of followers (internalization), the stronger the relationship will be between leader MI (internalization) and follower perceptions of ethical leadership.

Similarly to ethical leadership, perceptions of LMX quality are defined as a descriptive evaluation of the leader-follower relationship (Graen and Uhl-Bien, 1995). Therefore and in addition to the interactive effect of leader and follower MI on ethical leadership perceptions, we also propose that the interaction between leader and follower MI extends to follower perceptions of LMX quality. More precisely, because leader MI (internalization) and associated moral actions invite perceptions of ethical leadership especially for followers with a stronger MI (internalization), leader MI will invite a more positive perception of the quality of LMX for followers who are higher in MI.

**FIGURE 1:**
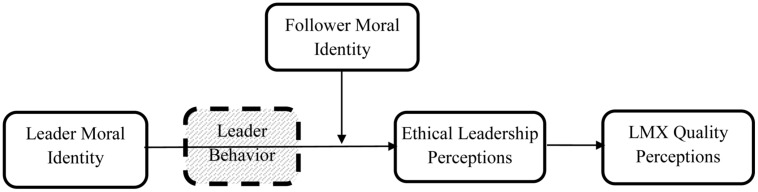
**Moderated mediation model.** The dotted box represents an unmeasured explanatory mechanism.

Note that we do not assume that follower MI moderates the relationship between followers’ perceptions of ethical leadership and LMX quality. We rather assume that both of these constructs are based on perceptions grounded in the normatively appropriate conduct of the leader – which is, in our perspective, defined by followers’ MI centrality. Consequently, for followers high in MI (internalization), leader MI (internalization) should positively influence perceptions of LMX quality via perceptions of ethical leadership. In contrast, followers low in MI should perceive the leader to be less ethical because they notice ethical behavior cues less due to its relatively lower relevance and salience. As a consequence, high leader MI (and the implied behavior that follows) should not translate into higher perceptions of LMX for followers low in MI. Therefore, we hypothesize that the interactive effect between leader and follower MI on the perceptions of ethical leadership will influence the perceived quality of LMX. **Figure 1** graphically displays this moderated mediation hypothesis.

H2: Follower MI moderates the relationship between leader MI and perceived quality of LMX via ethical leadership perceptions: follower perceptions of ethical leadership mediate the relationship between leader MI and perceived quality of LMX for followers with a stronger (vs. weaker) MI.

## Materials and Methods

### Sample and Level of Analysis

Because our predictions focus on the relationship between leaders and followers, we collected dyadic data to test our hypotheses. The final sample comprised 101 dyads from multiple organizations across the Netherlands, varying in size and scope from small national enterprises to large multinationals. The team leaders worked at different levels of the organization and the companies were active in a range of industries. The team leaders were primarily men (72.3%), with an average age of 37.95 years (SD = 11.42). The leaders had on average 8.47 years of leadership experience (SD = 7.16). Followers were about equally divided in terms of gender (52.5% women), with an average age of 29.89 years (SD = 10.36). Most of the dyads worked in non-manufacturing industries (91.1%) and had worked together for an average of 3.33 years (SD = 4.00).

### Procedure

To recruit a sample from a wide range of industries and occupations, we made use of the established snowball sampling technique (Morgeson and Humphrey, 2006; Mayer et al., 2009; Zapata et al., 2013). Data were collected via masters students enrolled in a class at a large Dutch business school (for similar procedures see Skarlicki and Folger, 1997; Grant and Mayer, 2009; Mayer et al., 2012b). As part of a masters course requirement, each of the 52 students was asked to collect data from two dyads within his or her personal network, resulting in 104 dyads. Each student was provided with a packet including questionnaires and envelopes.

At the time the study was conducted, the business school did not have an official ethical review board. Therefore, the study has no official approval of the protocol. However, the study conforms to the recommendations of the ERIM internal review board (i.e., an ethics committee of the Rotterdam School of Management which was established after the study has been conducted). Further, the study was not invasive and does not involve any manipulations or measures that could affect participants in their well-being, self-esteem, or mood. In addition, there was no deception involved. Care was taken of the privacy and confidentiality of all participants. Finally, participants were *a priori* informed about the aim of the study and allowed to stop the study at any point in time.

Due to missing values on some of the measures, the sample used to test the hypotheses consisted of 101 dyads. Further, there was no overlap in leaders or followers between the dyads (i.e., this was part of the requirement). The questionnaires included a cover letter in which leaders and followers were informed of the objectives and procedure for the paper and pencil study and assured that all data collected during the survey would be treated anonymously. Individual codes on the questionnaires made it possible to match leaders to their followers. Followers completed measures of ethical leadership, MI, LMX quality, and demographic data, whereas leaders completed measures of MI and demographics^1^.

### Leader Survey

We measured MI using Aquino and Reed’s (2002) ten-item scale. Respectively, five items measured the sub-dimensions of symbolization and internalization. In this measure, participants are presented with a set of nine adjectives (e.g., caring, compassionate, fair, kind) that might represent particular characteristics of a person. Subsequently, participants rate the degree to which these characteristics represent an important part of their own identity using a scale ranging from 1 (=completely disagree) to 7 (=completely agree). The means, SD, correlations, and reliabilities of all the measures are presented in **Table 1**. Finally, leaders provided demographic details relating to their tenure as a leader, age, and organizational industry (1 = manufacturing, 2 = non-manufacturing; cf. Van Dyne and LePine, 1998).

**Table 1:** Means, SD, Cronbach’s alpha (in correlation matrix diagonal) and correlations of the variables.

	*M*	SD	(1)	(2)	(3)	(4)	(5)	(6)	(7)
(1) Leader moral identity (MI) internalization	5.86	0.74	(0.74)						
(2) Follower MI internalization	5.82	0.80	0.38^∗∗^	(0.75)					
(3) Leader MI symbolization	4.38	1.03	0.58^∗∗∗^	0.26^∗∗^	(0.78)				
(4) Follower MI symbolization	4.18	0.95	0.27^∗∗^	0.45^∗∗∗^	0.32^∗∗^	(0.75)			
(5) Ethical leadership	3.75	0.58	0.36^∗∗∗^	0.25^∗^	0.31^∗∗^	0.12	(0.84)		
(6) LMX quality	3.66	0.61	0.26^∗∗^	0.09	0.15	-0.003	0.64^∗∗∗^	(0.83)	
(7) Length of dyadic relationship	3.33	4.00	-0.02	0.12	-0.17	0.04	0.27^∗∗^	0.27^∗∗^	-

*^∗^p < 0.05; ^∗∗^p < 0.01; ^∗∗∗^p < 0.001. Moral Identity (MI) scores ranged from 1 to 7; ethical leadership and LMX quality ranged from 1 to 5; length of dyadic relationship in years.*

### Follower Survey

*Moral identity*, with its sub-dimensions of internalization and symbolization, was measured using Aquino and Reed’s (2002) 10-item scale. *Ethical leadership* was measured with the 10-item Ethical Leadership Scale (e.g., “My leader disciplines employees who violate ethical standards.”) developed by Brown et al. (2005), which ranges from 1 (strongly agree) to 5 (=strongly disagree). *LMX quality* was assessed using Graen and Uhl-Bien’s (1995) LMX7-scale (e.g., “How would you characterize your working relationship with your leader?”), which also uses a five-point scale. Finally, followers reported their age, gender, and the length of time they had spent working with their current leader (in years).

## Results

### Preliminary Analyses

A confirmatory factor analysis was performed to examine the distinctiveness of the ethical leadership and LMX quality scales because both were rated by the followers. A one-factor solution showed a moderate fit, χ^2^ (119) = 253.85, *p* < 0.001, CFI = 0.94, SRMR = 0.11. However, the expected two-factor model fitted the data well, χ^2^ (118) = 182.58, *p* < 0.001, CFI = 0.96, SRMR = 0.08 (see Kline, 1998), and significantly better than the one-factor model, Δχ^2^ (1) = 71.27, *p* < 0.001.

### Main Analyses

Based on our theoretical reasoning, we differentiated between the internalization and symbolization dimensions of MI and used the internalization dimension as independent variable. To test our hypotheses and model, we made use of an approach proposed by Hayes (2013). This procedure makes it possible to test specific predictions of moderated mediation. We used Model 7 of Hayes’ (2013) PROCESS utility for SPSS with 10,000 bootstrap samples, testing exactly the type of mediated moderation suggested by our hypotheses, depicted in **Figure 1**. The model (i.e., Analysis 1 in **Table 2**) was tested in three steps. First, for the mediator variable model, a regression analysis was conducted to predict the mediator variable (i.e., ethical leadership) from the independent (i.e., leader and follower MI internalization) variables and their interaction. Leader and follower MI symbolization as well as length of dyadic relationship were added as control variables. We controlled for dyadic relationship tenure because previous research showed that it is a strong predictor of perceptions of leadership in dyadic relationships (Wayne et al., 1997, 2002) due to the mere-exposure effect (i.e., we start to like things more the more we are exposed to them; Zajonc, 1968). In this step of the analysis, we would expect a statistically significant interaction effect – supporting H1. Second, for the dependent variable model, the mediator (i.e., ethical leadership), independent variable (i.e., leader MI internalization), and control variables are regressed on the dependent variable. The mediator should be significant. The third and final step tests specific mediation effects. More precisely, statistical analyses test the indirect effect of leader MI on LMX quality via ethical leadership at different levels of the moderator (i.e., follower MI internalization). Bootstrapping is used to test mediation (Efron and Tibshirani, 1993). This analysis includes the control variables. Steps 2 and 3 are both tests of H2. Additionally, we ran the same analysis without leader and follower MI symbolization as control variables to show that the findings are not due to a suppression effect. The results are reported as Analysis 2 (see **Table 2**).

**Table 2:** Moderated mediation analysis with LMX quality perceptions as dependent variable, ethical leadership as mediator, leader MI internalization as independent variable, and follower MI internalization as moderator.

	Analysis 1					Analysis 2				
	*b*	SE *b*	*t*	95% *CI*		*b*	SE *b*	*t*	95% *CI*	
				Lower	Upper				Lower	Upper
**Mediator model**					
Length of dyad relationship	0.04	0.01	3.34^∗∗^	0.02	0.07	0.04	0.01	2.96^∗∗^	0.01	0.06
Leader MI symbolization	0.13	0.06	1.99^∗^	0.0002	0.25					
Follower MI symbolization	-0.04	0.06	-0.58	-0.16	0.09					
Leader MI internalization	0.17	0.09	1.93	-0.005	0.34	0.26	0.04	3.45^∗∗∗^	0.11	0.41
Follower MI internalization	0.13	0.08	1.63	-0.03	0.29	0.13	0.08	1.69	-0.02	0.28
Interaction leader x follower MI internalization	0.15	0.07	2.29^∗^	0.02	0.28	0.16	0.07	2.36^∗^	0.02	0.29

**Dependent variable model**					
Length of dyad relationship	0.02	0.01	1.22	-0.01	0.04	0.02	0.01	1.35	-0.01	0.04
Leader MI symbolization	-0.03	0.06	-0.48	-0.140	0.09					
Follower MI symbolization	-0.05	0.05	-1.11	-0.16	0.05					
Leader MI internalization	0.08	0.08	1.03	-0.08	0.24	0.04	0.07	0.62	-0.09	0.18
Ethical leadership perceptions	0.63	0.09	6.77^∗∗∗^	0.45	0.82	0.62	0.09	6.81^∗∗∗^	0.44	0.80

	**Indirect effect**		**SE**	**95% CI**		**Indirect effect**		**SE**	**95% CI**	
				**lower**	**upper**				**Lower**	**Upper**

**Conditional indirect effects at levels of follower MI internalization**					
High (+1SD)	0.18		0.08	0.05	0.37	0.24		0.08	0.08	0.40
Low (-1SD)	0.03		0.07	-0.11	0.20	0.08		0.07	-0.05	0.23

*Leader and follower MI symbolization served as control variables in Analysis 1. Length of dyadic relationship served as control variable in both analyses.*

*^∗^p < 0.05; ^∗∗^p < 0.01; ^∗∗∗^p < 0.001. MI scores were centralized (Aiken and West, 1991). Ninety-five percent confidence intervals are given for b-values. MI, moral identity.*

**FIGURE 2:**
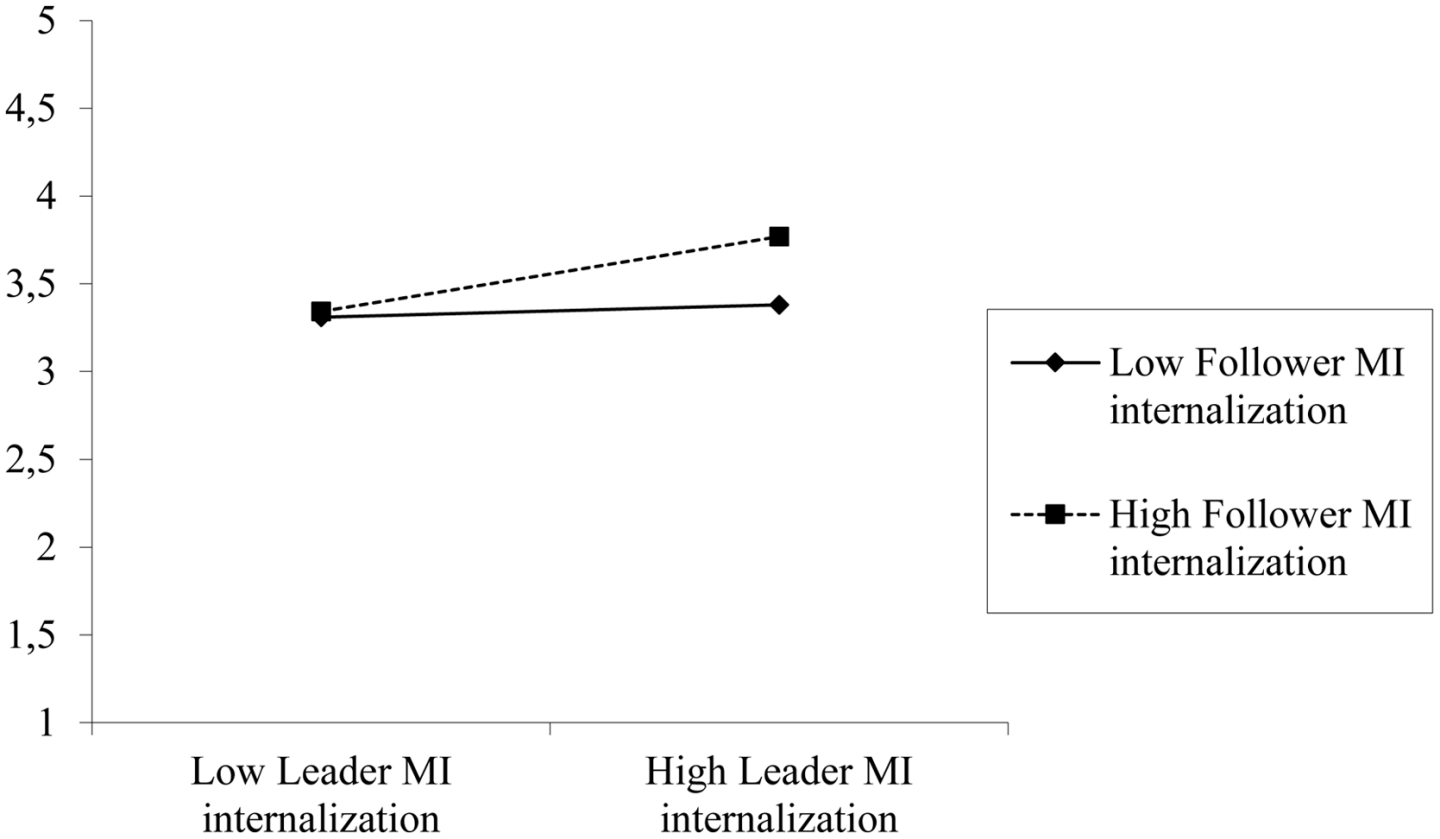
**Interactive effects of follower and leader MI (moral identity) internalization on ethical leadership perceptions based on Analysis 1 reported in **Table 2****.

As shown in **Table 2**, the mediator model yielded a statistically significant interaction between leader and follower MI internalization (i.e., with and without controlling for MI symbolization). The interaction effect (Analysis 1) is plotted in **Figure 2** and shows that leader MI internalization only relates positively to followers’ perceptions of ethical leadership if followers have stronger MI internalization, *b* = 0.29, SE = 0.10, 95% CI [0.08, 0.49], *p* = 0.006, but not if they have lower levels of MI internalization, *b* = 0.05, SE = 0.10, 95% CI [–0.15, 0.25], *p* = 0.64. Thus, this step provides support for H1. The dependent variable model yielded a statistically significant effect of ethical leadership perceptions on LMX quality perceptions, while the main effect of leader MI internalization is non-significant. Finally, the indirect effect analyses with bootstrapping indicate that mediation is stronger and statistically significant for high but not for low follower MI (i.e., 0.18 vs. 0.03; see **Table 2**). This is in line with H2 – suggesting that the stronger the follower MI internalization, the stronger is the mediating effect from leader MI via perceptions of ethical leadership on perceived quality of LMX.

### *Post Hoc* Analyses

We also ran some *post hoc* analyses with leader and follower MI symbolization as independent variables. Although the interaction effect of the MI symbolization dimension on ethical leadership perceptions was in the same direction as the effects reported for the internalization dimension, the effects did not reach conventional levels of significance. In addition, we ran analyses with an overall measure of MI (i.e., averaging all 10 items on the MI scale). These analyses yielded statistical significant effects as predicted by our hypotheses. Finally, we ran *post hoc* analyses on leaders’ performance evaluations (see footnote 1) of their followers’ (i.e., helping behavior, voice behavior, in-role performance; Van Dyne and LePine, 1998) as dependent variables (i.e., instead of followers’ perceptions of LMX quality). The analyses on helping behavior and voice behavior also provided statistically significant results for our model. While the effect on in-role performance was not statistically significant, a similar but weaker pattern of results was evident. The complete analyses can be requested from the first author. All of these analyses are reported in the supplementary material, because these were not the primary, *a priori* focus of our research.

## Discussion

The current research integrates and extends previous research on the effects of MI and follower perceptions of ethical leadership. Previous research indicated that stronger leader MI results in stronger perception that the leader is behaving ethically (Mayer et al., 2012a) which in turn influences perceptions of LMX quality (Mahsud et al., 2010; Tumasjan et al., 2011), because of the moral actions of leaders for whom MI is an important part of their self-concept (Reed and Aquino, 2003; Aquino et al., 2007; Reynolds and Ceranic, 2007). The current research extends this perspective by showing that this relationship is bounded by follower MI. Taking a relational perspective on leadership (Dansereau et al., 1975; Graen and Scandura, 1987; Graen and Uhl-Bien, 1995; Dulebohn et al., 2012; Jordan et al., 2013), the main contribution of the present research is to show that leader MI only has a real effect on whether followers perceive the leader to be behaving ethically when those followers themselves have a strong MI. In other words, leader MI does not predict ethical leadership perceptions for all followers. Rather, a precondition seems to be that followers have a high MI. In this way, the current research establishes that perceptions of ethical leadership are best predicted from a relational perspective on leadership instead of a leader perspective alone. In turn, such perceptions of ethical leadership relate to perceptions of LMX quality (Mahsud et al., 2010; Tumasjan et al., 2011).

### Theoretical and Practical Implications

Moral identity has been conceptualized as having two sub-dimensions: internalization and symbolization (Aquino and Reed, 2002). We assumed that the internalization dimension should be a stronger predictor of potential behavior by leaders, because powerful persons are more immune to situational pressures and act more upon their internalized value orientation (Galinsky et al., 2008). Furthermore, we also expected that followers’ internalization of MI would moderate the effects between leaders’ internalization of MI and perceptions of ethical leadership, because this facet is more central to followers’ self-concept (Aquino and Reed, 2002) and is therefore more likely to color followers’ perceptions of the leader (Markus, 1977; Lord and Maher, 1991; Van Quaquebeke et al., 2011). Although our results provide support for these assumptions, we do not aim to argue that the symbolization dimension plays no role in the predicted relationship. Indeed our additional analyses (see Supplementary Material) indicate that the effects are similar, but weaker. An interesting question is under what conditions might these effects of symbolization gain in strength. We may speculate that the sense of power (Anderson et al., 2012) a leader actually experiences may play a moderating role on the link between the MI dimensions and the leader’s actual behavior. A stronger sense of power should be related to behavior based on an internalized value-system, because leaders feel very comfortable with acting out what they feel to be (morally) right (Galinsky et al., 2008). In contrast, when leaders feel little power, they may feel more inclined to act in accordance with what others feel to be appropriate. Consequently, their behavior should be influenced more strongly by MI symbolization. While it was not the goal of the current research to test this assumption, future research might examine conditions under which the symbolization of a leader’s MI plays a stronger role in shaping followers’ perceptions of ethical leadership and, consequently, their perceptions of LMX quality.

The current results also indicate that the interaction between leader and follower MI on perceptions of ethical leadership translates into follower perceptions of LMX quality. More precisely, leader MI exerts its influence on LMX quality via ethical leadership perceptions especially for followers for whom MI is very central. These findings may be thus especially valuable for contexts in which followers have a high MI. In contrast, for followers low in MI, leader MI does not translate into perceived ethical leadership and LMX as easily. This leads to a qualification of the assumption behind LMX theory that “good” leadership should translate into high LMX. One possible reason is that followers low in MI might not pick up the cues from ethical leaders as much, as they do not consider them relevant to their identity. One possible solution for organizations might be to establish a culture with strong ethical values, an ethical mandate, and/or formal or informal ethical infrastructures (cf. Eisenbeiß and Giessner, 2012). In such cases, the leader’s MI is likely to translate better into followers’ perceptions of ethical leadership, because the increased emphasis makes it easier to notice, and hence should also improve the quality of the leader–follower relationships.

Leaders who define themselves strongly in terms of moral traits are more likely to behave consistently in ways that fit with this moral sense of self, but this does not imply that all followers will have the same perception of the ethicality of such behavior and, as a result, will judge the quality of their relationship with the leader to be equally positive. Thus, our results extend the perspective on ethical leadership by defining it as a perceptional phenomenon that is rooted in the relationship between leader and follower. This is especially important when we are focusing on the question of what followers perceive as ethical leadership. MI and the moral actions it elicits have been defined in terms of moral traits like honesty, kindness, and compassion (Aquino and Reed, 2002). We do not assume that low MI implies having no morality or having low expectations of morality. Rather, in line with the original definition of MI as a parameter of social identity (Markus, 1977; Tajfel and Turner, 1986; Aquino and Reed, 2002), we argue that low MI simply indicates that a person does not connect moral traits such as honesty, kindness, and compassion with his or her self-concept. Consequently, for these persons other aspects of their self-concept might be salient when they are judging leader behaviors (cf. Lord and Maher, 1991; Van Quaquebeke et al., 2011). Extending this idea, individuals might differ in the traits they consider to represent morality, and might therefore disagree about what behaviors or characteristics qualify as moral and which are thus useful to judge the ethicality of a leader. This is reflected in the ongoing philosophical discussion dating back to Plato and Aristotle (Northouse, 2010). Northouse (2010), for instance, differentiates three moral perspectives: ethical egoism, utilitarianism, and altruism. The first reflects the view that an individual strives for the best results for herself or himself (Avolio and Locke, 2002). The second aims “to create the greatest good for the greatest number” (Northouse, 2010, p. 379). Finally, altruism refers to moral behavior whose primary purpose is to help others. MI seems to have the strongest overlap with the concept of altruism. Hence, if followers have other conceptions of morality, the behavior of leaders who are high in MI (as defined by Aquino and Reed, 2002) should not have any effect on their perception of ethicality. For instance, if followers value utilitarianism, they might base their judgment of ethical leadership not on a leader’s altruistic behavior, but in how far that leader maximizes specific utilities with an action (e.g., laying off an employee in order to secure the jobs of all other employees). Our research supports this view, and we may speculate that followers low in MI might have other moral conceptualizations and, as a result, might use different moral schema when judging what makes the leader ethical (cf. Giessner and Van Quaquebeke, 2010). Therefore, it might be interesting to study other types of self-defined moral traits and how these relate to followers’ own self-definitions in future research. A promising approach was, for instance, laid out by Giessner and Van Quaquebeke (2010); they outline how “normatively appropriate conduct” can be defined in ethical leadership theory using Fiske’s (2004) idea of four discrete relational models that dictate what is considered the right mode of exchange. One of these relational models is communal sharing, which overlaps with the concept of MI as defined by Aquino and Reed (2002) in the sense that it also places emphasis on caring and altruism. Studying the other types of morality in the relationship between leaders and followers (i.e., authority ranking, equality matching, and market pricing) might therefore be a valuable avenue for future research.

### Caveats and Limitations

When discussing potential theoretical and practical implications, one has to be aware that our study is not without its weaknesses and limitations. First, we developed our predictions in a causal order. However, field studies, and especially cross-sectional ones, do not allow for testing of causality. Thus, it may be possible that there are different causal relationships between the constructs we have focused on than are suggested in the current paper. In spite of this potential limitation, our predictions and findings are in line with previous theoretical reasoning (Brown and Treviño, 2006) and empirical findings (Tumasjan et al., 2011; Mayer et al., 2012a). Nevertheless, future research might try to develop an experimental set-up with mundane realism that would enable our model to be tested.

Second, although we reasoned that leader MI results in leader behavior which will in turn influence follower perceptions, we did not measure actual behavior. While our assumption is grounded in previous research on the effects of MI (Aquino and Reed, 2002; Reed and Aquino, 2003; Reynolds and Ceranic, 2007), we believe that it would be valuable for future research to attempt to measure behavior objectively. Nevertheless, our results illustrate that ethical leadership as measured in the current study provides “only” a subjective evaluation of followers – not reflecting any actual behavior (Brown et al., 2005). Therefore, the interpretation of such a measurement in future studies should be in terms of subjective evaluations instead of behavioral manifestations.

Related to the previous point, our data are partly biased by common method biases (Podsakoff et al., 2003), because we made use of one common method (i.e., questionnaires). While we measured leader and follower MI via different sources, we believe that a behavioral measure on leader behavior could further reduce the common method bias in future research. To reduce the common method bias for our two outcome variables (i.e., LMX quality and ethical leadership perceptions), may be, however, more difficult. Theoretically, both measures are subjective perceptions of followers. Consequently, both are best measured by asking followers directly. One option might be to include a time-lag in the measurement model (Podsakoff et al., 2003).

Another limitation might be that our data could still be nested within contexts or situations which we did not measure (e.g., the organization). While we took care that data were not nested within the leader, the possibility of other higher-order nesting variables cannot be excluded. Furthermore, we should note that data have been collected via students. Consequently, we did not have fully control of the data collection process. While the procedure has been used in many previous studies (e.g., Skarlicki and Folger, 1997; Grant and Mayer, 2009; Mayer et al., 2012b), we have to recognize this limitation. Finally, we used a rather heterogeneous sample. However, because in this case the extraneous variables should produce more variation, a sample of this kind should reduce the chances of being able to confirm our hypotheses. Therefore, one might consider the sample heterogeneity as a strength of our study. Nevertheless, it might also be worthwhile testing our model with a more homogeneous sample.

## Conclusion

This study is important in demonstrating that the effect of leader MI on perceptions of ethical leadership is not as straightforward as it may at first appear. As a function of their MI, some followers are more sensitive to leader morality than others. Moreover, this translates to perceptions of LMX quality, which is crucially important for leadership effectiveness and follower satisfaction. The current study thus extends an invitation to researchers to treat ethical leadership not (purely) as an objective given phenomenon but rather for what it is – namely, something that exists at least in part in the eye of the beholder. That might not make the research itself simpler but it might make it more accurate in its predictions.

## Supplementary material

The Supplementary Material for this article can be found online at: http://journal.frontiersin.org/article/10.3389/fpsyg.2015.01126

## Conflict of Interest Statement

The authors declare that the research was conducted in the absence of any commercial or financial relationships that could be construed as a potential conflict of interest.

## Footnotes

1 The survey included additional measures that were used for course purposes only (i.e, a session on leadership research and development). None of these measures have been used in any publication, nor are there plans to use them. Additional measures in the survey: *Followers*: leader performance: helping, voice and in-role performance (Van Dyne and LePine, 1998); leader effectiveness (Giessner and van Knippenberg, 2008); leader group prototypicality (van Knippenberg and van Knippenberg, 2005); team identification (Mael and Ashforth, 1992); moral awareness (Reynolds, 2006); an exemplary four-item measure for 360° feedback; *Leaders*: follower performance: helping, voice and in-role performance (Van Dyne and LePine, 1998); accountability to the team (Giessner et al., 2013); leader group prototypicality (van Knippenberg and van Knippenberg, 2005); an exemplary four-item measure for 360° feedback.
