# Supplementary material for: In the moral eye of the beholder: the interactive effects of leader and follower moral identity on perceptions of ethical leadership and LMX quality
Source: Front Psychol. 2015 Aug 4;6:1126. doi: 10.3389/fpsyg.2015.01126 (PMC4523705; doi:10.3389/fpsyg.2015.01126)
Supplement: Supplementary file 1 [file Supplementary_Analyses.DOCX]

**Supplement - Post-hoc analyses**

We also ran additional post-hoc analyses with leader and follower moral identity symbolization as independent variables. In one analysis, we treated moral identity internalization and length of dyadic relationship as control variables, and in another we left control variables out (see Table 1). Although the interaction effect of the MI symbolization dimension on ethical leadership perceptions was in the same direction as the effects reported for the internalization dimension, the effects did not reach conventional levels of significance.

In addition, we ran analyses with an overall measure of moral identity (i.e., averaging all 10 items on the moral identity scale). These analyses yielded statistical significant effects as predicted by our hypotheses (see Table 2).

Finally, we ran post-hoc analyses on leaders’ performance evaluations of their followers’ (i.e., helping behavior, voice behavior, in-role performance) as dependent variables (i.e., instead of followers’ perceptions of LMX quality). As we did not predict these effects *a priori*, we only report these analyses here. The analyses on helping behavior and voice behavior also provided statistically significant results for our model. While the effect on in-role performance was not statistically significant, a similar but weaker pattern of results was evident (see Table 3).

Table 1. Hierarchical Regression Analysis of Moral Identity Symbolization on Ethical Leadership

|  | Analysis 1 | | | | | |  | Analysis 2 | | | | |
| --- | --- | --- | --- | --- | --- | --- | --- | --- | --- | --- | --- | --- |
|  | *b* | *SE b* | *t* | | *95 % CI* | |  | *b* | *SE b* | *t* | *95 % CI* | |
|  |  |  |  |  | *lower* | *upper* |  |  |  |  | *lower* | *upper* |
| *Mediator model (predicting ethical leadership perceptions)* |  | | | | | | | | | | | |
| Length of dyad relationship | .05 | .01 | 3.41 ** | | .02 | .07 |  | .05 | .01 | 3.72 *** | .02 | .08 |
| Leader MI symbolization | .13 | .06 | 1.96 | | -.002 | .25 |  | .20 | .05 | 3.66 *** | .09 | .31 |
| Follower MI symbolization | -.05 | .06 | -.87 | | -.18 | .07 |  | -.01 | .06 | -.19 | -.13 | .10 |
| Leader MI internalization | .16 | .09 | 1.77 | | -.02 | .33 |  |  |  |  |  |  |
| Follower MI internalization | .09 | .08 | 1.21 | | -.06 | .25 |  |  |  |  |  |  |
| Interaction leader x  follower MI symbolization | .08 | .05 | 1.64 | | -.02 | .17 |  | .06 | .05 | .11.39 | -.0. | .16 |
|  |  | | |  |  |  |  |  |  |  |  |  |
| *Note.* * *p* < .05; ** *p* < .01; *** *p* < .001. Moral identity symbolization scores were centralized. 95% Confidence intervals are given for *b* values. | | | | | | | | | | | | |

Table 2. Hierarchical Regression Analysis of Overall Moral Identity on Ethical Leadership

|  |  | | | | Ethical Leadership | | |  |
| --- | --- | --- | --- | --- | --- | --- | --- | --- |
|  | *b* | *SE b* | β |  | | 95 % CI | |  |
|  |  |  |  | *t* | | lower | upper |  |
| *Step 1* |  | | | |  | | |  |
| Length of dyad relationship | .04 | .01 | .27 ** | 2.81 | | .012 | .067 |  |
| *Step 2* |  | | | |  | | |  |
| Length of dyad relationship | .05 | .01 | .32 ** | 3.54 | | .020 | .072 |  |
| Leader moral identity | .30 | .07 | .40 *** | 4.08 | | .152 | .438 |  |
| Follower moral identity | .01 | .08 | .01 | .13 | | -.141 | .161 |  |
| *Step 3* |  | | | |  | | |  |
| Length of dyad relationship | .05 | .01 | .34 *** | 3.80 | | .024 | .075 |  |
| Leader moral identity | .29 | .07 | .38 *** | 3.93 | | .140 | .422 |  |
| Follower moral identity | .05 | .08 | .07 | .68 | | -.101 | .205 |  |
| Leader moral identity X  follower moral identity | .14 | .07 | .20 * | 2.15 | | .011 | .276 |  |
| *Note.* * *p* < .05; ** *p* < .01; *** *p* < .001. Moral identity scores were centralized. 95% Confidence intervals are given for *b* values. | | | | | | | | |

Table 3. Mediation Analysis on helping behavior, voice behavior and in-role performance

|  | Helping behavior | | | |  | Voice Behavior | | | |  | In-Role Performance | | | |
| --- | --- | --- | --- | --- | --- | --- | --- | --- | --- | --- | --- | --- | --- | --- |
|  | *b* | *SE* | *t* | |  | *b* | *SE* | *t* | |  | *b* | *SE* | *t* | |
| *Dependent variable model* |  |  |  | |  |  |  |  | |  |  |  |  | |
| Length of dyad relationship | -.01 | .01 | -.93 | |  | -.00 | .01 | -.34 | |  | .01 | .02 | .72 | |
| Leader MI symbolization | .08 | .06 | 1.36 | |  | .20 | .06 | 3.17 ** | |  | .01 | .07 | .20 | |
| Follower MI symbolization | -.04 | .06 | -.74 | |  | .004 | .06 | .07 | |  | -.08 | .07 | -1.19 | |
| Leader MI internalization | .01 | .08 | .13 | |  | -.13 | .08 | -1.60 | |  | .07 | .10 | .68 | |
| Ethical leadership  perceptions | .25 | .10 | 2.52 * | |  | .29 | .10 | 2.99 ** | |  | .22 | .12 | 1.91 | |
|  |  |  |  | |  |  |  |  | |  |  |  |  | |
| *Conditional indirect effects at levels of follower moral identity internalization* | *IE* | *SE* | *95% CI* | |  | *IE* | *SE* | *95% CI* | |  | *IE* | *SE* | *95% CI* | |
|  |  |  | *L* | *U* |  |  |  | *L* | *U* |  |  |  | *L* | *U* |
| High (+1SD) | .07 | .04 | .01 | .17 |  | .08 | .04 | .02 | .19 |  | .06 | .05 | -.005 | .18 |
| Low (-1SD) | .01 | .03 | -.04 | .09 |  | .01 | .03 | -.04 | .09 |  | .01 | .03 | -.04 | .09 |

*Note.* * *p* < .05; ** *p* < .01; *** *p* < .001. Moral identity scores were centralized. 95% Confidence intervals are given for *b* values.
